# Supplementary material for: Overcoming the dichotomy between open and isolated populations using genomic data from a large European dataset
Source: Sci Rep. 2017 Feb 1;7:41614. doi: 10.1038/srep41614 (PMC5286425; doi:10.1038/srep41614)
Supplement: Supplementary Information [file srep41614-s1.pdf]

Overcoming the dichotomy between open and isolated populations using genomic data from a large European dataset

Paolo Anagnostou, Valentina Dominici, Cinzia Battaggia, Luca Pagani, Miguel Vilar, Spencer Wells, Davide Pettener, Stefania Sarno, Alessio Boattini, Paolo Francalacci, Vincenza Colonna, Giuseppe Vona, Carla Calò, Giovanni Destro Bisol and Sergio Tofanelli

Content

Text S1. Information on population history

Text S2. Power of the set of SNPs in detecting signals of isolation.

Figure S1. (A) Gaussian kernel density estimation of the RoH size distribution in all populations. (B) Inferred assignment of RoH into the six classes and relative boundaries (in Kb).

Figure S2. Boxplots of the average number of RoHs in each size class among open and isolated populations. In brackets the boundaries for each RoH size in Kb. M-W stands for Mann-Whitney U test.

Figure S3. Boxplots of (A) number of RoHs, (B), total lenght of RoHs and (C) percentage of homozygous genotypes at the populat on level.

Figure S4. Violin plots of (A) Intra-population pairwise IBS sharing (B) Pairwise IBD blocks sharing at the population level.

Figure S5. Boxplots of the average number of RoHs in the six size classes at the population level.

Figure S6. Scatter plot of the genetic and geographic distances (in km). The regression lines between all populations and only for open populations are black dashed and blue continuous, respectively.

Figure S7. Principal components analysis performed on SNP data (A) First and second principal components (B) Third and fourth principal components.

Figure S8. Maximum likelihood estimation of individual ancestries for the 24 populations analysed in this study for K= 2-4.

Table S1. Average values and 95% confidence intervals of 100 randomly resampled populations without replacing for Sappada and Croatia.

Table S2. Population pairwise ratios between standard deviations of homozygosity at the individual level. Green and red cells indicate ratios higher and lower than 1, respectively. N.S. stands for not significant Levene test for equality of variances. Abbreviations as in Table 1.

Table S3. Population pairwise ratios between standard deviations of average pairwise IBS at the individual level. Green and red cells indicate ratios higher and lower than 1, respectively. N.S. stands for not significant Levene test for equality of variances. Abbreviations as in Table 1.

Table S4. Population pairwise ratios between standard deviations of number of RoHs at the individual level. Green and red cells indicate ratios higher and lower than 1, respectively. N.S. stands for not significant Levene test for equality of variances. Abbreviations as in Table 1.

Table S5. Population pairwise ratios between standard deviations of total length of RoHs at the individual level. Green and red cells indicate ratios higher and lower than 1, respectively. N.S. stands for not significant Levene test for equality of variances. Abbreviations as in Table 1.

Table S6. Population pairwise ratios between standard deviations of average sharing of IBD blocks at the individual level. Green and red cells indicate ratios higher and lower than 1, respectively. N.S. stands for not significant Levene test for equality of variances. Abbreviations as in Table 1.

Table S7. Values of the number and total length of RoHs obtained with stringent and relaxed settings. Abbreviations as in Table 1.

Table S8. Effective population size estimates based on 68205 snps (16 chromosomes). Abbreviations as in Table 1.

Table S9. Estimates of current effective population size using two different methods and SNP panels. The values obtained with the IBD method for BAS and RUS refer to 333 ybp whereas for ORK and FRA to 300 ybp. Abbreviations as in Table 1.

Table S10. Population pairwise ratios between values of number of generations necessary to reach the observed inbreeding coefficient at any given Ne. Upper and lower bounds were obtained assuming initial inbreeding coefficients to be equal to the highest and lowest values observed among open populations, respectively.

Table S11. Multiple regression analysis performed on the entire dataset and the isolated and open population groups separately.

SUPPLEMENTARY TEXT S1

German-speaking linguistic islands

The group of isolated population from the northeastern Italy include three linguistic islands (micro-isolates) of the Eastern Italian Alps (Sappada, Sauris and Timau) and a Cimbrian group from Eastern pre-Alps (Lessinia). Sappada is a municipality of 1307 inhabitants (ISTAT 2010) located at an altitude of 1245 m above sea level (a.s.l.) in the province of Belluno (Veneto region). According to historical records, this community was founded in the eleventh century AD from a small group coming East Tyrol (today Austrian district of Lienz)[1]. Sauris and Timau are two villages of the Carnic Alps in the province of Udine (Friuli Venezia Giulia region). The former has 429 inhabitants (ISTAT 2010), is located in the upper Lumiei valley (1212 m .a.s.l.) and its founders probably came from the lower Carinthia and North Tyrol in the thirteenth century AD[2]. Timau is a small village counting around 500 inhabitants, situated at 830 m a.s.l. in the But valley. It has been claimed that the community originated from two different migration events from the neighboring Austrian region of Carinthia in the eleventh and thirteenth century AD[3]. The first Cimbrian settlers probably came from Bavaria around the eleventh century AD and settled in the nearby mountainous areas of Asiago, Luserna/Lavarone and Lessinia in the province of Verona (Veneto region)[4]. This latter area is a mountainous territory (around 800-1000 m a.s.l.) on the border with Trentino and accounts for 13455 inhabitants (ISTAT 2010).

Previous studies, based on unilinear markers, have shown for these communities a certain degree of genetic isolation from the surrounding populations[5]. Analyses of genetic variation highlight low haplotype diversity and lack of any signal of demographic expansion, whereas, at inter-population level, Sauris, Sappada and Timau clearly differentiate from other European populations. An unexpected finding relates to the extreme genetic differentiation among Sappada, Sauris and Timau, considering their relative geographic proximity and closeness in terms of language and traditions. This seems to suggest that members of linguistic islands Eastern Alps do not self-identify as belonging to the same community despite their cultural homogeneity[6]. This “local ethnicity” may have played a role in marriage strategies. decreasing the genetic exchange among the three linguistic islands[5].

Sardinia

The whole Sardinia can be considered a genetic isolate because of geographical and cultural barriers that limited gene flow from other populations[7]. Several studies, based on both autosomal and uniparental markers have shown that the genome of current Sardinian inhabitants retains some signatures of a long history of isolation[8,9,10].

The North Sardinian samples were collected in villages from Barbagia of Nuoro, Goceano, Monte Acuto, a wide area characterized by the presence of high hills and mountains. Archeological data attest the presence of humans since Nuragic period (from ~3900 to ~2900 ybp)[11]. Located in the southwestern part of the Sardinia, Sulcis Iglesiente is one of the early populated area of the island. According to archaeological evidence, its first inhabitants occupied the area around 5000 years ago[12]. Phoenicians, Punics, Cartagines and Romans were later attracted by this area because of its rich mining compound[13].

Within the island, some geographically well localized populations can be identified. Carloforte is the only town located on the small island of San Pietro, which is off the southwestern coast of Sardinia (Italy), with a surface area of 50,24 km^2. Carloforte is an alloglot community founded in 1738 by Ligurian refugees coming from the Tunisian island of Tabarka. Even today, the spoken language is the ancient dialect of Pegli (Liguria). According to the 2011 census, the population of Carloforte reached 6,301 inhabitants.

Benetutti, a small village of 1971 inhabitants (Census 2011), is located at 406 m a.s.l. in an area of central-north Sardinia, the Goceano, characterized by a strong isolation[14]. Whereas archaeological remains testify its ancient origin (probably 5000 ybp)[14], first available demographic documents relative to this population (Quinque Libri) date back to 1618. Studying Y chromosome and mtDNA, it was possible to detect signatures of genetic isolation in Benetutti and Carloforte[15,16,17]. In fact, haplotype diversity observed in Carloforte and Benetutti are lower than the ones observed in neighbouring populations and their genetic distance from other Sardinian populations were found to be statistically significant. In particular, the MDS plot based on mtDNA evidence a peculiar behaviour of Carloforte which lies close to African populations, particularly Tunisia, possibly due to admixture with Tunisian females.

Other European isolates

Basques are settled in the western edge of the Pyrenees in the Spanish–French border. The language spoken in the Basque country (Euskara) has no proven relationships with any other languages now spoken in Europe or elsewhere[18,19]. To date, around 650,000 people still speak Basque (Arrizabalaga 2008). Historical and theonymic evidence suggests that a proto-basque language was spoken in this area by the time of Roman conquest[20]. Recent studies based on the application of NGS techniques on ancient and extant individuals revealed that Basques trace their genetic ancestry to early Iberian

farmers (from ~5,500 to ~3,500 ybp)[21].Besides their linguistic isolation, the uniqueness of Basques has been addressed by studying their gene pool. However, there is still no consensus on the isolation of the Basque population. A large number of genetic studies, based on mitochondrial DNA[22], Y

chromosome[23], and autosomal markers[24,25], consider Basques as a distinct genetic isolate. In complete contrast, other studies based on genomic diversity have shown that Basques fall well within the European genetic landscape[26,27].

The Orkney islands are situated off the north coast of Great Britain and is inhabited by 21,349 people (2011 census). Orcadians are currently regarded to as an isolated population due to the geographic position and the prohibitive sea conditions that make the islands difficult to reach[28]. The Orkney archipelago was first inhabited by Norse Vikings, between the late 8th and early 9th century[29], and the "Norwegian rule" lasted until 1472 when the islands passed under the Scottish control. A recent study on the fine-scale genetic structure of the United Kingdom highlighted the genetic difference between the Orkney and the rest of the English samples, which likely reflects both isolation and admixture outside UK[30].

Novel population data

The sample from North Italy was collected in the medium-sized city of Aosta (current census 35,000) located at an altitude of 583 m a.s.l. in the region of Valle d'Aosta. This city lies along one of the most important access routes to Northern Italy from North-Western and Central Europe. A relatively small portion of the Aosta population (around 15%) speaks the franco-provencal language (census 2011) also spread in a broad surrounding area including north-western Italy, France and Switzerland.

Albanian Gheg samples have been collected in the city of Tirana (current census 610,000) from healthy and unrelated volunteers, selected according to their self-declared affiliation to the wide Gheg linguistic group[31]. The Gheg dialect is traditionally spoken in the northern part of the country.

With the term Piana di Lucca is intended the area in Northern Tuscany extending eastwards from the city of Lucca (census size 2011: 87,200) to the Arno river (Altopascio, Capannori, Porcari, Montecarlo municipalities; census size 2011: 73,028). Today, it is a densely populated plain connected by a wide roadway network at the foot of a mountainous area (Apuan Alps, Apennines) which still retains ethno-linguistic[32] and genetic[33,17] features traceable to a pre-Latin cultural matrix.

References

1. Peratoner, A. *Sappada/Plodn. Storia, etnografia e ambiente naturale. Pieve di Cadore* (Tiziano Editore, 2002).
2. Brunettin, G. L'insediamento di Sauris tra storiografia e rappresentazione di un'origine in *Sauris, Zahre, una comunità delle Alpi carniche* (Cozzi, D., Isabella, D., Navarra, E., Eds) 43-6

(Forum Editrice Universita, 1998).

3. Petris, B. *Tischlbong Tamau Timau* (Del Bianco, 1980).
4. Rapelli, G. XIII comuni veronesi. La formazione dell'isola linguistica in *Isole di cultura* (Prezzi, 2004).
5. Capocasa, M. *et al.* Detecting genetic isolation in human populations: A study of European language minorities. *PLoS ONE.* **8,** e56371 (2013).
6. Steinicke, E., Walder, J., Löffler, R. & Beismann, M. Autochthonous linguistic minorities in the Italian Alps: *Revue de géographie alpine.* (2012). doi:10.4000/rga.1454
7. Francalacci, P. *et al.* Peopling of three Mediterranean islands (Corsica, Sardinia, and Sicily) inferred by y-chromosome biallelic variability. *Am J Phys Anthropol.* **121,** 270–279 (2003).
8. Di Gaetano, C. *et al.* Sardinians genetic background explained by runs of Homozygosity and Genomic regions under positive selection. *PLoS ONE.* **9,** e91237 (2014).
9. Pardo, L. M. *et al.* Dissecting the genetic make-up of north-east Sardinia using a large set of haploid and autosomal markers. *Eur J Hum Genet.* **20,** 956–964 (2012).
10. Piras, I. S. *et al.* Genome-wide scan with nearly 700 000 SNPs in two Sardinian sub-populations suggests some regions as candidate targets for positive selection. *Eur J Hum Genet.* **20,** 1155–1161 (2012).
11. Lilliu, G., Moravetti, A. *Arte E Religione Della Sardegna Prenuragica: Idoletti, Ceramiche, Oggetti D'Ornamento* (C. Delfino, 1999).
12. Atzeni, E. *La preistoria del Sulcis-Iglesiente* (STEF, 1987).
13. Bartoloni, P. *Fenici e cartaginesi nel Sulcis-Iglesiente* (C. Delfino, 2013).
14. Oppes, T. *Il goceano* (Edisar, 1990).
15. Robledo, R. *et al.* Analysis of a genetic isolate: The case of Carloforte (Italy). *Hum Biol.* **84,** 735–754 (2012).
16. Calò, C. M., Corrias, L., Vona, G., Bachis, V. & Robledo, R. Sampling strategies in a linguistic isolate: Results from mtDNA analysis. *Am J Hum Biol.* **24,** 192–194 (2012).
17. Capocasa, M. *et al.* Linguistic, geographic and genetic isolation: a collaborative study of Italian populations. *J Anthropol Sci.* **92**, 201–231 (2014).
18. Campbell, G. L. *Concise compendium of the world's languages*. (Taylor & Francis, 1998).
19. Comrie, B. & Aitchison, J. *The Atlas of languages*. (Facts On File, 2003).
20. Martinez-Cruz, B. *et al.* Evidence of Pre-Roman tribal genetic structure in Basques from Uniparentally inherited markers. *Mol Biol Evol.* **29,** 2211–2222 (2012).
21. Günther, T.*et al.* Ancient genomes link early farmers from Atapuerca in Spain to modern-day Basques. *Proc Natl Acad Sci USA.* **112,** 11917–11922 (2015).
22. García, O. *et al.* Using mitochondrial DNA to test the hypothesis of a European post-glacial human recolonization from the Franco-Cantabrian refuge. *Heredity* **106,** 37–45 (2010).
23. Chikhi, L., Nichols, R. A., Barbujani, G. & Beaumont, M. A. Y genetic data support the neolithic

demic diffusion model. *Proc Natl Acad Sci USA.* **99,** 11008–11013 (2002).

24. Pérez-Miranda, A. M. *et al.* Microsatellite data support subpopulation structuring among Basques. *J Hum Genet.* **50,** 403–414 (2005).
25. Rodríguez-Ezpeleta, N. *et al.* High-density SNP genotyping detects homogeneity of Spanish and french Basques, and confirms their genomic distinctiveness from other European populations. *Hum Genet.* **128,** 113–117 (2010).
26. Garagnani, P. *et al.* Isolated populations as treasure troves in genetic epidemiology: The case of the Basques. *Eur J Hum Genet.* **17,** 1490–1494 (2009).
27. Laayouni, H., Calafell, F. & Bertranpetit, J. A genome-wide survey does not show the genetic distinctiveness of Basques. *Hum Genet.* **127,** 455–458 (2010).
28. McQuillan R. *et al.* Runs of homozygosity in European populations. Am J Hum Genet. **83,** 359-372 (2008).
29. Davies, S. F. N. & Davies, N. *The Isles: A history*. (Oxford University Press, USA, 2001).
30. Leslie, S. *et al.* The fine-scale genetic structure of the British population. *Nature.* **519,** 309–314 (2015).
31. Sarno, S. *et al.* Shared language, diverging genetic histories: high-resolution analysis of Y-chromosome variability in Calabrian and Sicilian Arbereshe. Eur J Hum Genet. **24**,600-606 (2016).
32. Armanini M. Ligures apuani. Lunigiana storica, Garfagnana e Versilia prima dei romani. (Libreriauniveristaria.it, Italy, 2015)
33. Bertoncini S. *et al.* A Y Variant Which Traces the Genetic Heritage of Ligures Tribes. J Biol Res. **84**: 143-146 (2012).

Supplementary note S2

To test the power of our set of of SNPs (reduced panel, 87,818 SNPs) in detecting signals of isolation we compared them with more dense SNP data (complete panel, 647.789 SNPs) relative to the four HGDP populations which are also included in our dataset. The comparisons were performed using two measures of intra-population variation: Runs of Homozygosity and intra-population pairwise IBS identities as these statistics were found to be among the most informative to detect isolation signals (see main text). Figure 1 shows the distributions of the total length and number of RoHs found in the four HGDP populations using the same settings as in the main analyses. The portion of genome covered by RoHs was found to be from 5 to 10 times higher using the complete rather than the reduced SNPs panel (see table 1). Similarly, the average number of RoHs per individual recorded with the complete SNP panel was from 14 to 10 times higher than that observed using the reduced panel.

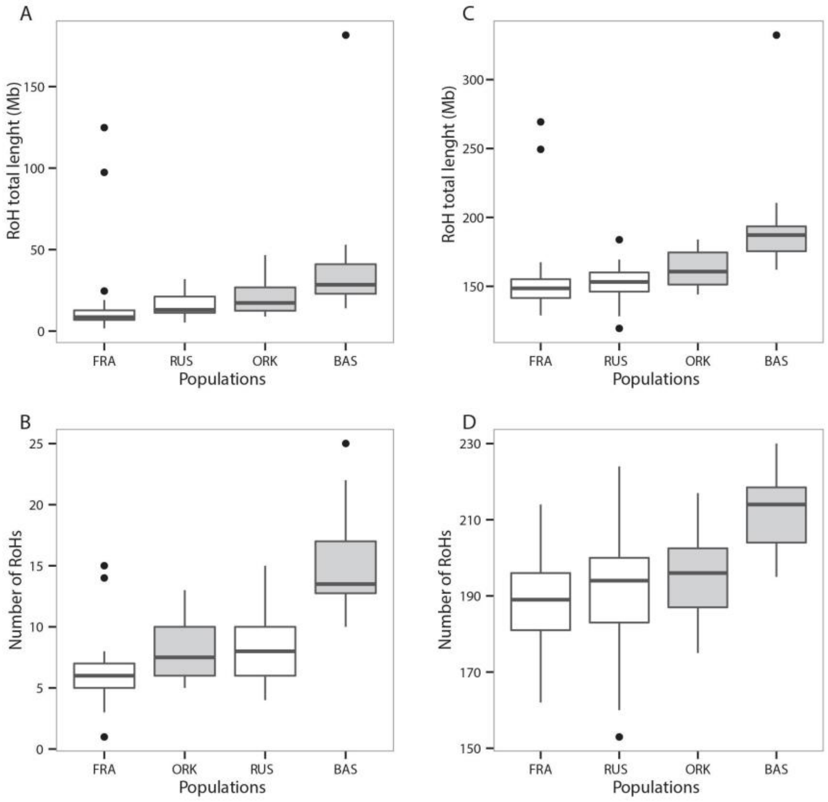

Figure 1. Distributions of the total length and number of RoH found in the four HGDP populations using the reduced loci set (87,818 SNPs), frames A and B, and the complete loci set (647,789 SNPs), frames C and D.

Table 1. Mean and standard deviations (s.d.) of the distributions of the total length (expressed in Mb) and number of RoHs discovered using the complete and reduced SNPs panels.

	Complete SNP panel				Reduced SNP panel			
	Number of RoHs		Total length of RoHs		Number of RoHs		Total length of RoHs	
	Average	s.d.	Average	s.d.	Average	s.d.	Average	s.d.
BAS	212.000	10.460	191.203	32.092	15.083	3.968	37.572	31.853
FRA	189.276	11.225	154.986	29.897	6.172	2.817	16.668	26.370
ORK	196.375	11.396	162.736	13.303	8.188	2.480	21.167	11.271
RUS	190.520	14.026	152.727	13.040	8.080	2.652	15.789	7.294

Despite the values obtained with the two SNP panels were sharply different in absolute terms, patterns of isolation with the reduced SNP panel remained substantially unchanged, with the median values of BAS and ORK always exceeding the values of FRA and RUS.

Moving to the distributions of the intra-population pairwise IBS identities (figure 2) we found that the values in absolute terms are slightly higher using the complete SNP panel. However, the two isolated populations show higher average values that open ones independently of the SNP panel used. Furthermore, with both the SNP panels, the distributions of isolated populations exhibit the upper tail indicative of the occurrence of a current or past high levels of inbreeding. However, it is worth noting that the extent of the tail was found to be smaller in BAS using the complete SNP panel.

On the whole, these analyses evidence that, despite a reduced number of markers, signals of genetic isolation detected by using the GenoChip 2.0 are fully comparable with those of the HGDP panel.

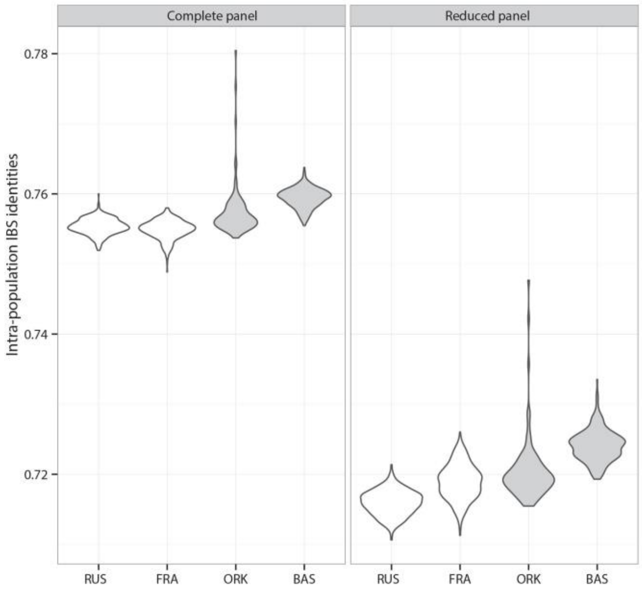

Figure 2. Violin plots of the distributions of intra-population pairwise IBS identities in the four populations analysed using the complete and reduced SNP panels.

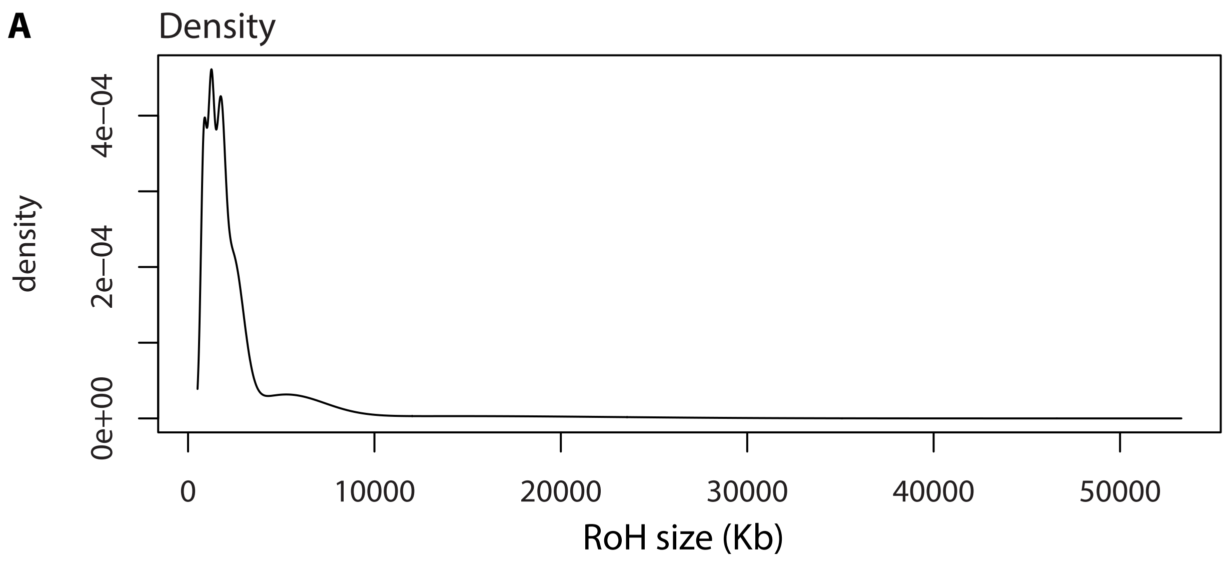

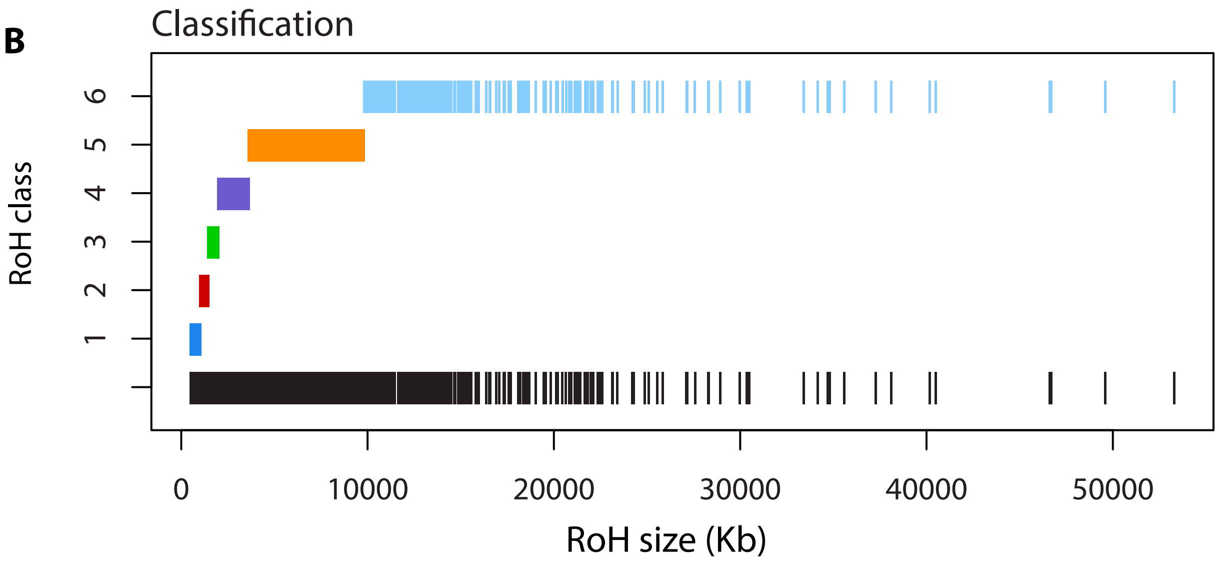

RoH size classes	Start	End
RoH class 1	0	1142.524
RoH class 2	1142.524	1328.346
RoH class 3	1328.346	2442.571
RoH class 4	2442.571	4016.658
RoH class 5	4016.658	7416.594
RoH class 6	7416.594	15025.38

Figure S1. (A) Gaussian kernel density estimation of the RoH size distribution in all populations. (B) Inferred assignment of RoH into the six classes and relative boundaries (in Kb).

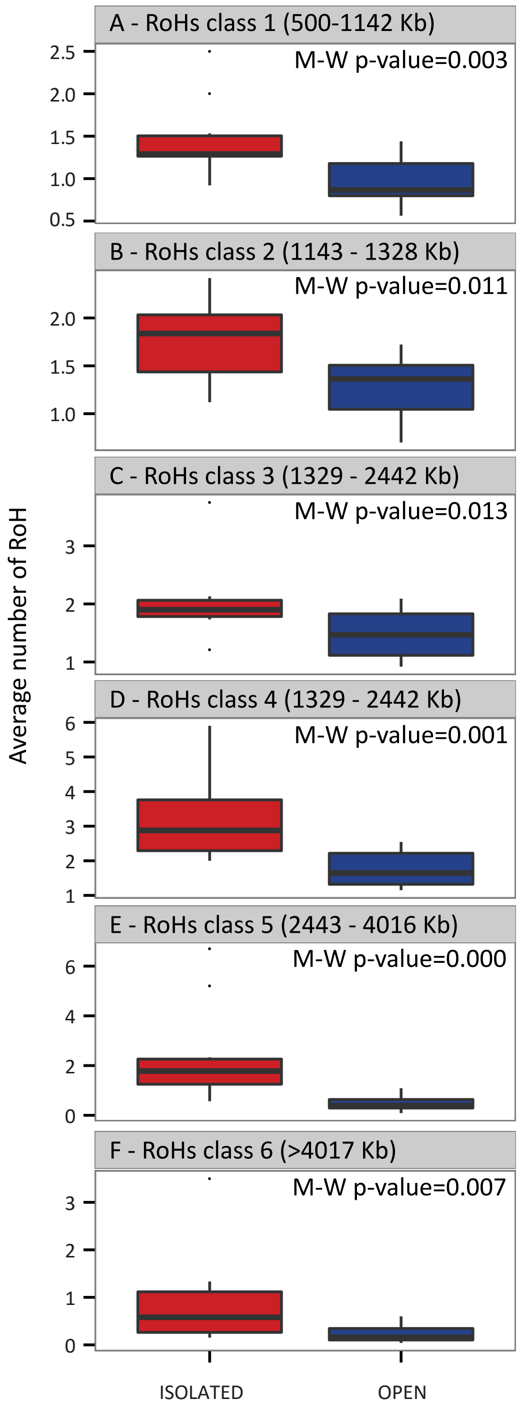

Figure S2. Boxplots of the average number of RoHs in each size class among open and isolated populations. In brackets the boundaries for each RoH size in Kb. M-W stands for Mann-Whitney U test.

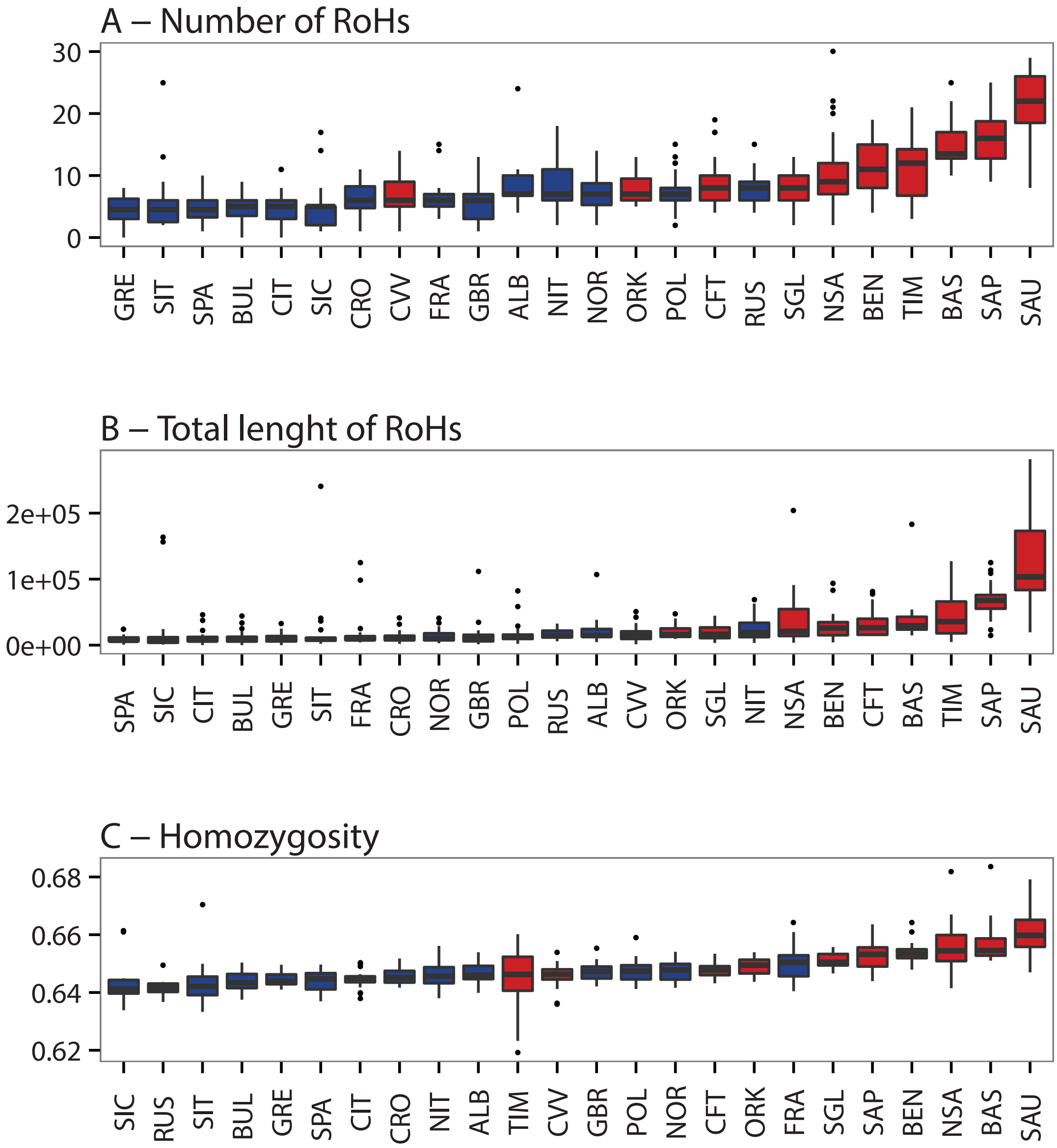

Figure S3. Boxplots of (A) number of RoHs, (B), total lenght of RoHs and (C) percentage of homozygous genotypes at the populat on level.

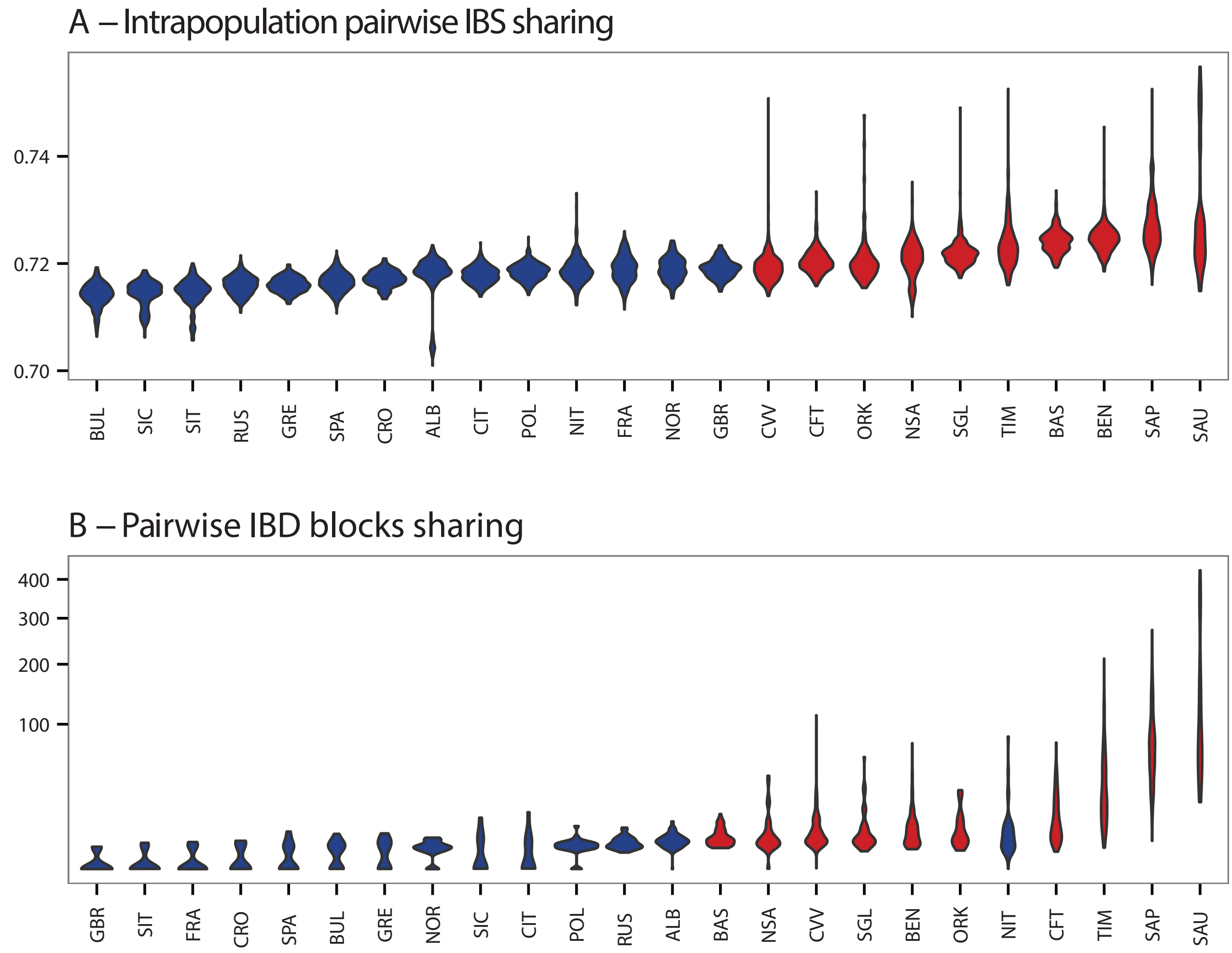

Figure S4. Violin plots of (A) Intra-population pairwise IBS sharing (B) Pairwise IBD blocks sharing at the population level.

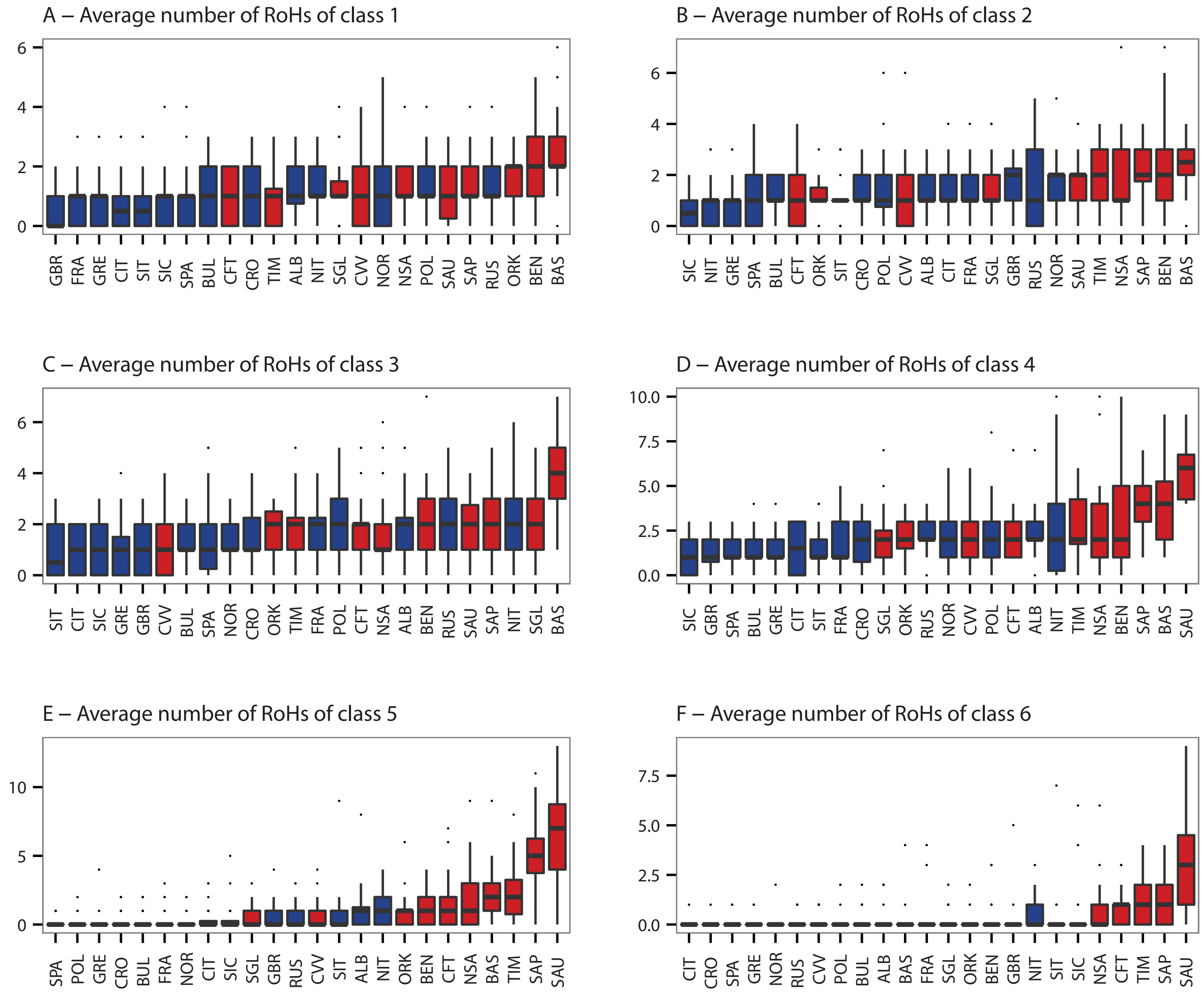

Figure S5. Boxplots of the average number of RoHs in the six size classes at the population level.

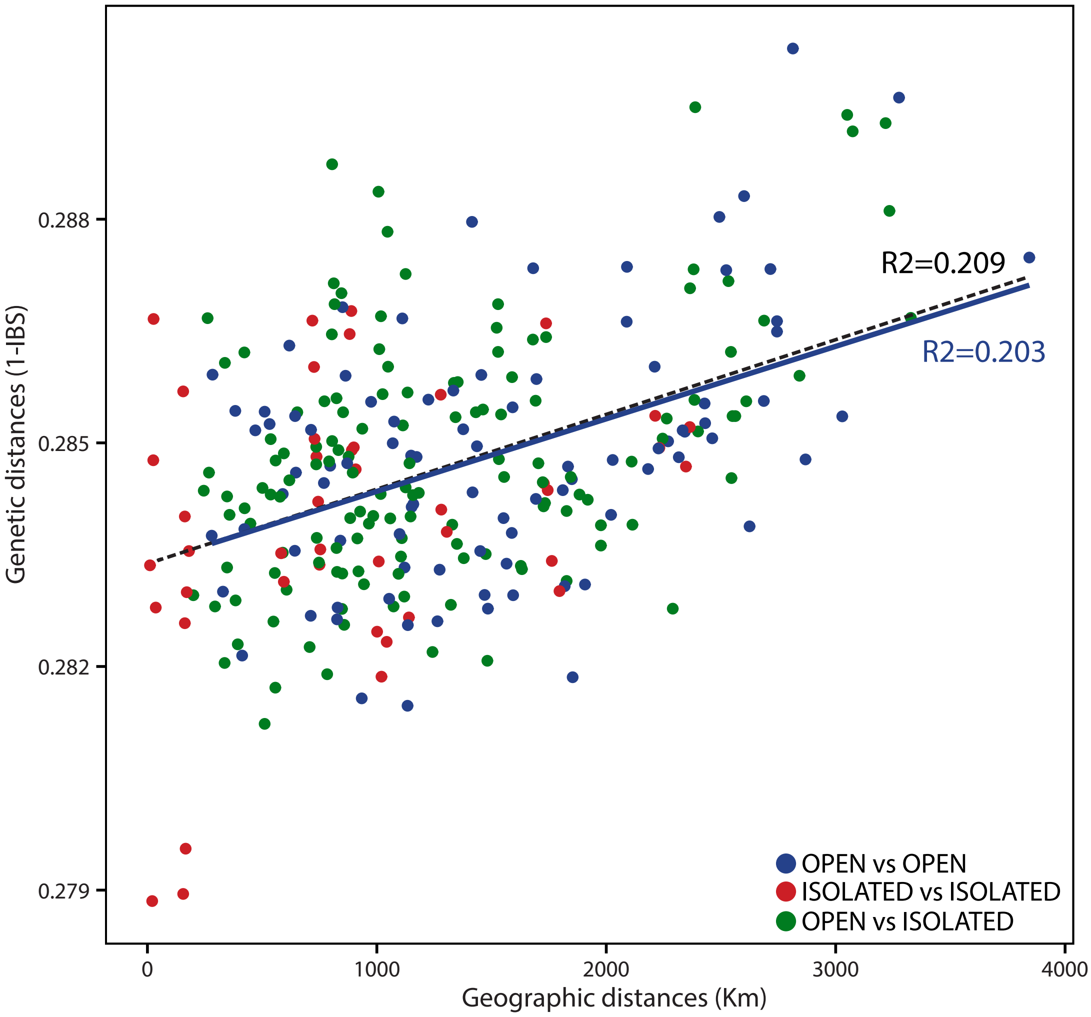

Figure S6. Scatter plot of the genetic and geographic distances (in km). The regression lines between all populations and only for open populations are black dashed and blue continuous, respectively.

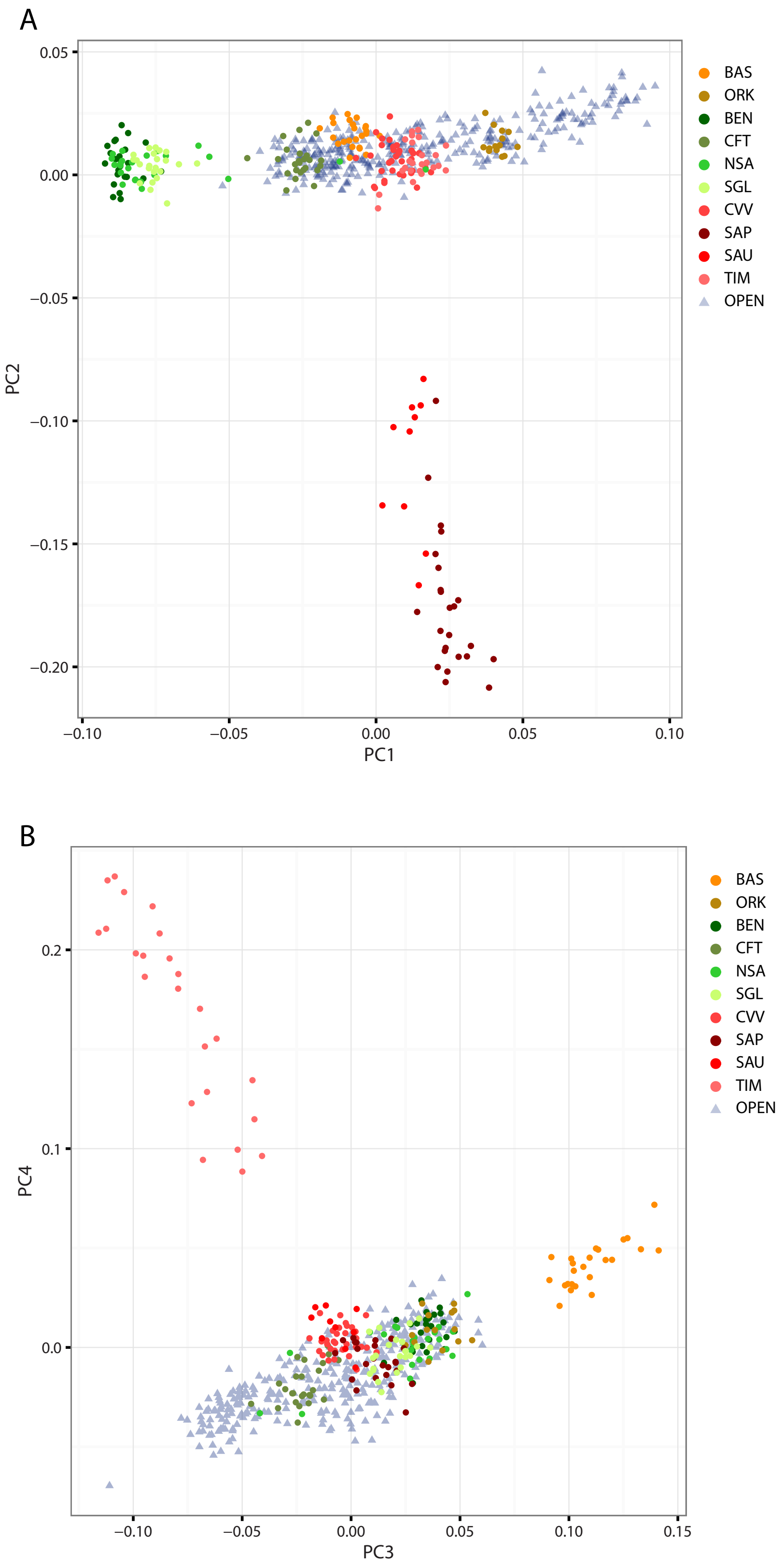

Figure S7. Principal components analysis performed on SNP data (A) First and second principal components (B) Third and fourth principal components.

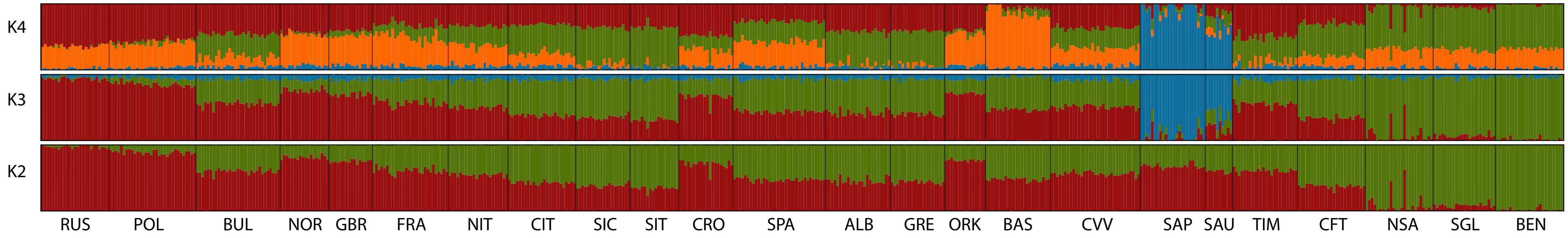

Figure S8. Maximum likelihood estimation of individual ancestries for the 24 populations analysed in this study for K= 2-4.

Table S1. Average values and 95% confidence intervals of 100 randomly resampled populations without replacing for Sappada and Croatia.

	Sample size		RoH average number	RoH average total length (kb)	Homozygosity
Sauris	10		21.200	130786.400	0.663
Sappada	24		16.125	68042	0.655
	10 (x100 resamplings)	Average	16.158	67849	0.655
		95% confidence interval	(13.995-18.410)	(54222-81207)	(0.653-0.657)
Croatia	20		6.050	12443	0.647
	10 (x100 resamplings)	Average	6.147	12140	0.647
		95% confidence interval	(4.900-7.200)	(7965-16042)	(0.646-0.648)

Table S2. Population pairwise ratios between standard deviations of homozygosity at the individual level. Green and red cells indicate ratios higher and lower than 1, respectively. N.S. stands for not significant Levene test for equality of variances. Abbreviations as in Table 1.

	BAS	BEN	CFT	CVV	NSA	ORK	SAP	SAU	SGL	TIM	ALB	BUL	CIT	CRO	FRA	GBR	GRE	NIT	NOR	POL	RUS	SIC	SIT	SPA
**BAS**	1.000	0.632	0.592	0.543	N.S.	N.S.	N.S.	2.197	0.578	1.860	N.S.	N.S.	0.301	N.S.	N.S.	N.S.	0.518	N.S.	N.S.	N.S.	0.478	N.S.	N.S.	N.S.
**BEN**	1.581	1.000	N.S.	N.S.	2.356	N.S.	1.693	3.475	N.S.	2.942	N.S.	N.S.	0.476	N.S.	1.805	N.S.	N.S.	N.S.	N.S.	N.S.	N.S.	N.S.	N.S.	N.S.
**CFT**	1.690	N.S.	1.000	N.S.	2.518	N.S.	1.810	3.714	N.S.	3.144	N.S.	N.S.	0.509	N.S.	1.930	N.S.	N.S.	1.613	N.S.	N.S.	N.S.	N.S.	1.701	1.353
**CVV**	1.843	N.S.	N.S.	1.000	2.745	1.424	1.973	4.048	N.S.	3.428	1.492	1.516	0.554	N.S.	2.103	N.S.	N.S.	1.758	1.546	N.S.	N.S.	N.S.	1.855	1.475
**NSA**	N.S.	0.424	0.397	0.364	1.000	N.S.	N.S.	N.S.	0.388	N.S.	0.543	0.552	0.202	0.454	N.S.	0.423	0.348	N.S.	N.S.	0.483	0.321	0.476	N.S.	0.537
**ORK**	N.S.	N.S.	N.S.	0.702	N.S.	1.000	N.S.	2.842	N.S.	2.406	N.S.	N.S.	0.389	N.S.	N.S.	N.S.	0.670	N.S.	N.S.	N.S.	0.618	N.S.	N.S.	N.S.
**SAP**	N.S.	0.591	0.553	0.507	N.S.	N.S.	1.000	2.052	0.540	1.738	N.S.	N.S.	0.281	N.S.	N.S.	N.S.	0.484	N.S.	N.S.	N.S.	0.446	N.S.	N.S.	N.S.
**SAU**	0.455	0.288	0.269	0.247	N.S.	0.352	0.487	1.000	0.263	N.S.	0.369	0.374	0.137	0.308	0.520	0.287	0.236	0.434	0.382	0.327	0.217	0.323	0.458	0.364
**SGL**	1.731	N.S.	N.S.	N.S.	2.579	N.S.	1.853	3.803	1.000	3.220	N.S.	N.S.	0.521	N.S.	1.976	N.S.	N.S.	N.S.	N.S.	N.S.	N.S.	N.S.	N.S.	N.S.
**TIM**	0.538	0.340	0.318	0.292	N.S.	0.416	0.576	N.S.	0.311	1.000	0.435	0.442	0.162	0.364	N.S.	0.338	0.278	0.513	0.451	0.387	0.257	0.381	N.S.	0.430
**ALB**	N.S.	N.S.	N.S.	0.670	1.840	N.S.	N.S.	2.714	N.S.	2.297	1.000	N.S.	0.372	N.S.	N.S.	N.S.	0.640	N.S.	N.S.	N.S.	0.590	N.S.	N.S.	N.S.
**BUL**	N.S.	N.S.	N.S.	0.660	1.811	N.S.	N.S.	2.670	N.S.	2.261	N.S.	1.000	0.366	N.S.	N.S.	N.S.	0.630	N.S.	N.S.	N.S.	0.581	N.S.	N.S.	N.S.
**CIT**	3.324	2.102	1.966	1.804	4.952	2.570	3.558	7.303	1.920	6.183	2.691	2.735	1.000	2.250	3.794	2.093	1.722	3.172	2.789	2.390	1.588	2.358	3.345	2.661
**CRO**	N.S.	N.S.	N.S.	N.S.	2.201	N.S.	N.S.	3.246	N.S.	2.749	N.S.	N.S.	0.445	1.000	1.687	N.S.	N.S.	N.S.	N.S.	N.S.	N.S.	N.S.	N.S.	N.S.
**FRA**	N.S.	0.554	0.518	0.475	N.S.	N.S.	N.S.	1.925	0.506	N.S.	N.S.	N.S.	0.264	0.593	1.000	0.552	0.454	N.S.	N.S.	0.630	0.419	N.S.	N.S.	N.S.
**GBR**	N.S.	N.S.	N.S.	N.S.	2.366	N.S.	N.S.	3.490	N.S.	2.954	N.S.	N.S.	0.478	N.S.	1.813	1.000	N.S.	N.S.	N.S.	N.S.	N.S.	N.S.	N.S.	N.S.
**GRE**	1.930	N.S.	N.S.	N.S.	2.876	1.492	2.067	4.241	N.S.	3.591	1.563	1.588	0.581	N.S.	2.203	N.S.	1.000	1.842	1.620	N.S.	N.S.	N.S.	1.943	1.545
**NIT**	N.S.	N.S.	0.620	0.569	N.S.	N.S.	N.S.	2.302	N.S.	1.949	N.S.	N.S.	0.315	N.S.	N.S.	N.S.	0.543	1.000	N.S.	N.S.	0.501	N.S.	N.S.	N.S.
**NOR**	N.S.	N.S.	N.S.	0.647	N.S.	N.S.	N.S.	2.618	N.S.	2.217	N.S.	N.S.	0.359	N.S.	N.S.	N.S.	0.617	N.S.	1.000	N.S.	0.569	N.S.	N.S.	N.S.
**POL**	N.S.	N.S.	N.S.	N.S.	2.072	N.S.	N.S.	3.056	N.S.	2.587	N.S.	N.S.	0.418	N.S.	1.588	N.S.	N.S.	N.S.	N.S.	1.000	0.665	N.S.	N.S.	N.S.
**RUS**	2.093	N.S.	N.S.	N.S.	3.118	1.618	2.240	4.598	N.S.	3.893	1.694	1.722	0.630	N.S.	2.389	N.S.	N.S.	1.997	1.756	1.505	1.000	N.S.	2.106	1.675
**SIC**	N.S.	N.S.	N.S.	N.S.	2.100	N.S.	N.S.	3.097	N.S.	2.622	N.S.	N.S.	0.424	N.S.	N.S.	N.S.	N.S.	N.S.	N.S.	N.S.	N.S.	1.000	N.S.	N.S.
**SIT**	N.S.	N.S.	0.588	0.539	N.S.	N.S.	N.S.	2.183	N.S.	N.S.	N.S.	N.S.	0.299	N.S.	N.S.	N.S.	0.515	N.S.	N.S.	N.S.	0.475	N.S.	1.000	N.S.
**SPA**	N.S.	N.S.	0.739	0.678	1.861	N.S.	N.S.	2.745	N.S.	2.324	N.S.	N.S.	0.376	N.S.	N.S.	N.S.	0.647	N.S.	N.S.	N.S.	0.597	N.S.	N.S.	1.000

Table S3. Population pairwise ratios between standard deviations of average pairwise IBS at the individual level. Green and red cells indicate ratios higher and lower than 1, respectively. N.S. stands for not significant Levene test for equality of variances. Abbreviations as in Table 1.

	BAS	BEN	CFT	CVV	NSA	ORK	SAP	SAU	SGL	TIM	ALB	BUL	CIT	CRO	FRA	GBR	GRE	NIT	NOR	POL	RUS	SIC	SIT	SPA
**BAS**	1.000	N.S.	N.S.	N.S.	1.601	2.006	2.152	4.880	N.S.	2.053	1.898	N.S.	0.719	0.677	1.112	0.756	0.634	N.S.	N.S.	0.701	0.776	N.S.	N.S.	0.800
**BEN**	N.S.	1.000	N.S.	N.S.	1.409	1.766	1.895	4.297	N.S.	1.807	1.672	N.S.	0.633	0.596	0.979	0.666	0.558	N.S.	N.S.	0.617	0.683	N.S.	N.S.	0.705
**CFT**	N.S.	N.S.	1.000	1.233	1.608	2.016	2.162	4.903	N.S.	2.062	1.908	N.S.	0.722	0.681	1.118	0.760	0.637	N.S.	N.S.	0.704	0.779	N.S.	1.210	0.804
**CVV**	N.S.	N.S.	0.811	1.000	1.305	1.635	1.754	3.977	N.S.	1.673	1.547	0.805	0.586	0.552	N.S.	0.616	0.517	N.S.	N.S.	0.571	0.632	N.S.	N.S.	0.652
**NSA**	0.625	0.710	0.622	0.767	1.000	N.S.	1.344	3.049	0.765	1.282	N.S.	0.617	0.449	0.423	0.695	0.472	0.396	0.715	0.598	0.438	0.485	0.673	0.753	0.500
**ORK**	0.498	0.566	0.496	0.612	N.S.	1.000	1.073	2.433	0.610	1.023	N.S.	0.492	0.358	0.338	0.554	0.377	0.316	0.570	0.477	0.349	0.387	0.537	0.600	0.399
**SAP**	0.465	0.528	0.462	0.570	0.744	0.932	1.000	2.268	0.569	N.S.	0.882	0.459	0.334	0.315	0.517	0.351	0.294	0.531	0.445	0.326	0.360	0.500	0.560	0.372
**SAU**	0.205	0.233	0.204	0.251	0.328	0.411	0.441	1.000	0.251	0.421	0.389	0.202	0.147	0.139	0.228	0.155	0.130	0.234	0.196	0.144	0.159	0.221	0.247	0.164
**SGL**	N.S.	N.S.	N.S.	N.S.	1.307	1.638	1.757	3.985	1.000	1.676	1.550	N.S.	0.587	0.553	0.908	0.618	0.518	N.S.	N.S.	0.572	0.634	N.S.	N.S.	0.653
**TIM**	0.487	0.553	0.485	0.598	0.780	0.977	N.S.	2.378	0.597	1.000	0.925	0.481	0.350	0.330	0.542	0.368	0.309	0.557	0.467	0.341	0.378	0.525	0.587	0.390
**ALB**	0.527	0.598	0.524	0.646	N.S.	N.S.	1.134	2.570	0.645	1.081	1.000	0.520	0.379	0.357	0.586	0.398	0.334	0.602	0.505	0.369	0.409	0.567	0.634	0.421
**BUL**	N.S.	N.S.	N.S.	1.242	1.620	2.031	2.178	4.940	N.S.	2.078	1.922	1.000	0.728	0.686	1.126	0.766	0.642	N.S.	N.S.	0.709	0.785	N.S.	1.219	0.810
**CIT**	1.391	1.580	1.384	1.707	2.226	2.790	2.993	6.788	1.703	2.855	2.641	1.374	1.000	N.S.	1.547	N.S.	0.881	1.591	1.332	N.S.	N.S.	1.498	1.675	1.113
**CRO**	1.476	1.677	1.469	1.811	2.363	2.962	3.177	7.205	1.808	3.030	2.803	1.458	N.S.	1.000	1.642	N.S.	N.S.	1.689	1.414	N.S.	1.145	1.590	1.778	1.181
**FRA**	0.899	1.021	0.895	N.S.	1.439	1.803	1.935	4.387	1.101	1.845	1.707	0.888	0.646	0.609	1.000	0.680	0.570	1.028	0.861	0.630	0.697	N.S.	N.S.	0.719
**GBR**	1.322	1.502	1.316	1.622	2.117	2.653	2.846	6.453	1.619	2.714	2.510	1.306	N.S.	N.S.	1.471	1.000	0.838	1.512	1.267	N.S.	N.S.	1.424	1.593	N.S.
**GRE**	1.578	1.792	1.570	1.936	2.526	3.165	3.396	7.700	1.932	3.239	2.996	1.559	1.134	N.S.	1.755	1.193	1.000	1.805	1.511	N.S.	1.224	1.699	1.901	1.263
**NIT**	N.S.	N.S.	N.S.	N.S.	1.399	1.754	1.882	4.267	N.S.	1.795	1.660	N.S.	0.629	0.592	0.973	0.661	0.554	1.000	N.S.	0.613	0.678	N.S.	N.S.	0.700
**NOR**	N.S.	N.S.	N.S.	N.S.	1.671	2.094	2.247	5.095	N.S.	2.143	1.982	N.S.	0.751	0.707	1.161	0.790	0.662	N.S.	1.000	0.731	0.810	N.S.	N.S.	0.835
**POL**	1.427	1.621	1.421	1.751	2.285	2.863	3.072	6.966	1.748	2.930	2.710	1.410	N.S.	N.S.	1.588	N.S.	N.S.	1.633	1.367	1.000	1.107	1.537	1.719	1.142
**RUS**	1.289	1.464	1.283	1.582	2.063	2.586	2.774	6.290	1.579	2.646	2.447	1.273	N.S.	0.873	1.434	N.S.	0.817	1.474	1.235	0.903	1.000	1.388	1.553	N.S.
**SIC**	N.S.	N.S.	N.S.	N.S.	1.487	1.863	1.999	4.532	N.S.	1.906	1.763	N.S.	0.668	0.629	N.S.	0.702	0.589	N.S.	N.S.	0.651	0.721	1.000	N.S.	0.743
**SIT**	N.S.	N.S.	0.826	N.S.	1.329	1.665	1.787	4.052	N.S.	1.704	1.576	0.820	0.597	0.562	N.S.	0.628	0.526	N.S.	N.S.	0.582	0.644	N.S.	1.000	0.664
**SPA**	1.250	1.419	1.244	1.533	2.000	2.507	2.690	6.099	1.530	2.565	2.373	1.235	0.899	0.847	1.390	N.S.	0.792	1.429	1.197	0.876	N.S.	1.346	1.505	1.000

Table S4. Population pairwise ratios between standard deviations of number of RoHs at the individual level. Green and red cells indicate ratios higher and lower than 1, respectively. N.S. stands for not significant Levene test for equality of variances. Abbreviations as in Table 1.

	BAS	BEN	CFT	CVV	NSA	ORK	SAP	SAU	SGL	TIM	ALB	BUL	CIT	CRO	FRA	GBR	GRE	NIT	NOR	POL	RUS	SIC	SIT	SPA
**BAS**	1.000	N.S.	0.619	N.S.	N.S.	N.S.	N.S.	1.907	N.S.	1.488	0.595	0.615	0.531	N.S.	0.435	N.S.	N.S.	N.S.	N.S.	0.516	0.652	0.582	0.592	0.618
**BEN**	N.S.	1.000	0.492	0.698	N.S.	0.547	N.S.	N.S.	N.S.	N.S.	0.473	0.489	0.423	0.617	0.346	N.S.	0.513	N.S.	N.S.	0.411	0.519	0.463	0.471	0.491
**CFT**	1.615	2.031	1.000	N.S.	N.S.	N.S.	2.108	3.080	N.S.	2.402	N.S.	N.S.	N.S.	N.S.	N.S.	N.S.	N.S.	2.014	N.S.	N.S.	N.S.	N.S.	N.S.	N.S.
**CVV**	N.S.	1.432	N.S.	1.000	N.S.	N.S.	1.486	2.171	N.S.	1.694	N.S.	N.S.	0.605	N.S.	0.495	N.S.	N.S.	N.S.	N.S.	0.588	N.S.	N.S.	N.S.	N.S.
**NSA**	N.S.	N.S.	N.S.	N.S.	1.000	N.S.	N.S.	1.973	N.S.	1.539	N.S.	N.S.	N.S.	N.S.	0.450	N.S.	N.S.	N.S.	N.S.	N.S.	N.S.	N.S.	N.S.	N.S.
**ORK**	N.S.	1.828	N.S.	N.S.	N.S.	1.000	1.898	2.772	N.S.	2.162	N.S.	N.S.	N.S.	N.S.	N.S.	N.S.	N.S.	1.813	N.S.	N.S.	N.S.	N.S.	N.S.	N.S.
**SAP**	N.S.	N.S.	0.474	0.673	N.S.	0.527	1.000	N.S.	N.S.	N.S.	0.456	0.471	0.407	0.594	0.333	N.S.	0.494	N.S.	N.S.	0.395	0.500	0.446	0.453	0.473
**SAU**	0.524	N.S.	0.325	0.461	0.507	0.361	N.S.	1.000	0.492	N.S.	0.312	0.322	0.279	0.407	0.228	0.456	0.338	N.S.	0.476	0.271	0.342	0.305	0.310	0.324
**SGL**	N.S.	N.S.	N.S.	N.S.	N.S.	N.S.	N.S.	2.034	1.000	1.587	N.S.	N.S.	0.567	N.S.	0.464	N.S.	N.S.	N.S.	N.S.	0.551	N.S.	N.S.	N.S.	N.S.
**TIM**	0.672	N.S.	0.416	0.590	0.650	0.462	N.S.	N.S.	0.630	1.000	0.400	0.413	0.357	0.521	0.292	0.585	0.434	N.S.	0.610	0.347	0.439	0.391	0.398	0.415
**ALB**	1.680	2.113	N.S.	N.S.	N.S.	N.S.	2.194	3.205	N.S.	2.500	1.000	N.S.	N.S.	N.S.	N.S.	N.S.	N.S.	2.096	N.S.	N.S.	N.S.	N.S.	N.S.	N.S.
**BUL**	1.626	2.045	N.S.	N.S.	N.S.	N.S.	2.123	3.102	N.S.	2.419	N.S.	1.000	N.S.	N.S.	N.S.	N.S.	N.S.	2.028	N.S.	N.S.	N.S.	N.S.	N.S.	N.S.
**CIT**	1.882	2.367	N.S.	1.653	N.S.	N.S.	2.457	3.589	1.764	2.800	N.S.	N.S.	1.000	N.S.	N.S.	N.S.	N.S.	2.347	N.S.	N.S.	N.S.	N.S.	N.S.	N.S.
**CRO**	N.S.	1.622	N.S.	N.S.	N.S.	N.S.	1.684	2.459	N.S.	1.919	N.S.	N.S.	N.S.	1.000	0.561	N.S.	N.S.	N.S.	N.S.	N.S.	N.S.	N.S.	N.S.	N.S.
**FRA**	2.300	2.893	N.S.	2.021	2.223	N.S.	3.004	4.387	2.156	3.422	N.S.	N.S.	N.S.	1.784	1.000	2.000	1.484	2.869	2.087	N.S.	1.501	N.S.	N.S.	N.S.
**GBR**	N.S.	N.S.	N.S.	N.S.	N.S.	N.S.	N.S.	2.193	N.S.	1.711	N.S.	N.S.	N.S.	N.S.	0.500	1.000	N.S.	N.S.	N.S.	0.594	N.S.	N.S.	N.S.	N.S.
**GRE**	N.S.	1.949	N.S.	N.S.	N.S.	N.S.	2.024	2.956	N.S.	2.306	N.S.	N.S.	N.S.	N.S.	0.674	N.S.	1.000	1.933	N.S.	N.S.	N.S.	N.S.	N.S.	N.S.
**NIT**	N.S.	N.S.	0.497	N.S.	N.S.	0.552	N.S.	N.S.	N.S.	N.S.	0.477	0.493	0.426	N.S.	0.349	N.S.	0.517	1.000	N.S.	0.414	0.523	0.466	0.475	0.495
**NOR**	N.S.	N.S.	N.S.	N.S.	N.S.	N.S.	N.S.	2.102	N.S.	1.640	N.S.	N.S.	N.S.	N.S.	0.479	N.S.	N.S.	N.S.	1.000	0.569	N.S.	N.S.	N.S.	N.S.
**POL**	1.936	2.436	N.S.	1.701	N.S.	N.S.	2.529	3.693	1.815	2.881	N.S.	N.S.	N.S.	N.S.	N.S.	1.684	N.S.	2.415	1.757	1.000	N.S.	N.S.	N.S.	N.S.
**RUS**	1.533	1.928	N.S.	N.S.	N.S.	N.S.	2.001	2.923	N.S.	2.280	N.S.	N.S.	N.S.	N.S.	0.666	N.S.	N.S.	1.912	N.S.	N.S.	1.000	N.S.	N.S.	N.S.
**SIC**	1.719	2.162	N.S.	N.S.	N.S.	N.S.	2.244	3.278	N.S.	2.557	N.S.	N.S.	N.S.	N.S.	N.S.	N.S.	N.S.	2.144	N.S.	N.S.	N.S.	1.000	N.S.	N.S.
**SIT**	1.689	2.125	N.S.	N.S.	N.S.	N.S.	2.206	3.222	N.S.	2.514	N.S.	N.S.	N.S.	N.S.	N.S.	N.S.	N.S.	2.107	N.S.	N.S.	N.S.	N.S.	1.000	N.S.
**SPA**	1.619	2.036	N.S.	N.S.	N.S.	N.S.	2.114	3.087	N.S.	2.408	N.S.	N.S.	N.S.	N.S.	N.S.	N.S.	N.S.	2.019	N.S.	N.S.	N.S.	N.S.	N.S.	1.000

Table S5. Population pairwise ratios between standard deviations of total length of RoHs at the individual level. Green and red cells indicate ratios higher and lower than 1, respectively. N.S. stands for not significant Levene test for equality of variances. Abbreviations as in Table 1.

	BAS	BEN	CFT	CVV	NSA	ORK	SAP	SAU	SGL	TIM	ALB	BUL	CIT	CRO	FRA	GBR	GRE	NIT	NOR	POL	RUS	SIC	SIT	SPA
**BAS**	1.000	N.S.	N.S.	0.646	2.259	N.S.	N.S.	7.199	N.S.	3.091	N.S.	0.458	0.346	0.473	0.340	0.537	0.510	N.S.	0.587	0.426	0.670	0.613	0.253	0.340
**BEN**	N.S.	1.000	N.S.	0.596	2.085	N.S.	N.S.	6.644	N.S.	2.853	0.661	0.423	0.320	0.437	0.314	0.496	0.471	N.S.	0.542	0.394	0.619	0.565	0.233	0.314
**CFT**	N.S.	N.S.	1.000	0.437	1.529	N.S.	N.S.	4.872	N.S.	2.092	0.485	0.310	0.234	0.320	0.230	0.363	0.345	N.S.	0.397	0.289	0.454	0.415	0.171	0.230
**CVV**	1.549	1.678	2.288	1.000	3.499	N.S.	2.080	11.150	N.S.	4.787	N.S.	0.709	0.536	N.S.	0.527	N.S.	N.S.	2.177	N.S.	0.660	N.S.	N.S.	0.391	0.527
**NSA**	0.443	0.480	0.654	0.286	1.000	0.392	0.595	3.187	0.436	N.S.	0.317	0.203	0.153	0.209	0.151	0.238	0.226	0.622	0.260	0.189	0.297	0.271	0.112	0.151
**ORK**	N.S.	N.S.	N.S.	N.S.	2.548	1.000	N.S.	8.122	N.S.	3.487	N.S.	0.516	0.391	0.534	0.384	N.S.	N.S.	1.585	N.S.	0.481	N.S.	N.S.	0.285	0.384
**SAP**	N.S.	N.S.	N.S.	0.481	1.682	N.S.	1.000	5.361	N.S.	2.302	N.S.	0.341	0.258	0.352	0.253	N.S.	0.380	N.S.	0.437	0.318	N.S.	0.456	0.188	0.253
**SAU**	0.139	0.151	0.205	0.090	0.314	0.123	0.187	1.000	0.137	0.429	0.100	0.064	0.048	0.066	0.047	0.075	0.071	0.195	0.082	0.059	0.093	0.085	0.035	0.047
**SGL**	N.S.	N.S.	N.S.	N.S.	2.292	N.S.	N.S.	7.305	1.000	3.136	N.S.	0.465	0.351	0.480	0.345	N.S.	0.518	N.S.	N.S.	0.433	N.S.	N.S.	0.256	0.345
**TIM**	0.324	0.351	0.478	0.209	N.S.	0.287	0.434	2.329	0.319	1.000	0.232	0.148	0.112	0.153	0.110	0.174	0.165	0.455	0.190	0.138	0.217	0.198	0.082	0.110
**ALB**	N.S.	1.512	2.063	N.S.	3.153	N.S.	N.S.	10.049	N.S.	4.314	1.000	0.639	0.483	0.660	0.475	N.S.	N.S.	1.962	N.S.	0.595	N.S.	N.S.	0.352	0.475
**BUL**	2.185	2.367	3.228	1.410	4.934	1.936	2.934	15.726	2.153	6.752	1.565	1.000	N.S.	N.S.	N.S.	N.S.	N.S.	3.070	N.S.	N.S.	1.464	N.S.	N.S.	N.S.
**CIT**	2.889	3.130	4.268	1.865	6.525	2.560	3.879	20.794	2.846	8.927	2.069	N.S.	1.000	N.S.	N.S.	1.551	N.S.	4.059	N.S.	N.S.	1.936	N.S.	N.S.	N.S.
**CRO**	2.115	2.291	3.124	N.S.	4.776	1.874	2.840	15.222	2.084	6.535	1.515	N.S.	N.S.	1.000	N.S.	N.S.	N.S.	2.971	N.S.	N.S.	N.S.	N.S.	N.S.	N.S.
**FRA**	2.938	3.183	4.341	1.897	6.636	2.604	3.945	21.149	2.895	9.080	2.105	N.S.	N.S.	N.S.	1.000	1.578	N.S.	4.129	N.S.	N.S.	1.969	N.S.	N.S.	N.S.
**GBR**	1.862	2.018	2.752	N.S.	4.207	N.S.	N.S.	13.407	N.S.	5.756	N.S.	N.S.	0.645	N.S.	0.634	1.000	N.S.	2.617	N.S.	N.S.	N.S.	N.S.	0.470	0.633
**GRE**	1.959	2.123	2.895	N.S.	4.426	N.S.	2.631	14.105	1.931	6.056	N.S.	N.S.	N.S.	N.S.	N.S.	N.S.	1.000	2.753	N.S.	N.S.	N.S.	N.S.	N.S.	N.S.
**NIT**	N.S.	N.S.	N.S.	0.459	1.607	0.631	N.S.	5.123	N.S.	2.199	0.510	0.326	0.246	0.337	0.242	0.382	0.363	1.000	0.418	0.303	0.477	0.436	0.180	0.242
**NOR**	1.704	1.846	2.517	N.S.	3.849	N.S.	2.288	12.266	N.S.	5.266	N.S.	N.S.	N.S.	N.S.	N.S.	N.S.	N.S.	2.394	1.000	N.S.	N.S.	N.S.	0.430	N.S.
**POL**	2.345	2.541	3.465	1.514	5.297	2.079	3.149	16.882	2.311	7.248	1.680	N.S.	N.S.	N.S.	N.S.	N.S.	N.S.	3.296	N.S.	1.000	1.572	N.S.	N.S.	N.S.
**RUS**	1.492	1.617	2.204	N.S.	3.370	N.S.	N.S.	10.741	N.S.	4.611	N.S.	0.683	0.517	N.S.	0.508	N.S.	N.S.	2.097	N.S.	0.636	1.000	N.S.	0.377	0.507
**SIC**	1.633	1.769	2.412	N.S.	3.688	N.S.	2.192	11.752	N.S.	5.046	N.S.	N.S.	N.S.	N.S.	N.S.	N.S.	N.S.	2.294	N.S.	N.S.	N.S.	1.000	0.412	0.555
**SIT**	3.960	4.291	5.851	2.557	8.945	3.510	5.318	28.509	3.903	12.240	2.837	N.S.	N.S.	N.S.	N.S.	2.127	N.S.	5.565	2.324	N.S.	2.654	2.426	1.000	N.S.
**SPA**	2.940	3.186	4.344	1.898	6.641	2.606	3.948	21.166	2.897	9.087	2.106	N.S.	N.S.	N.S.	N.S.	1.579	N.S.	4.132	N.S.	N.S.	1.971	1.801	N.S.	1.000

Table S6. Population pairwise ratios between standard deviations of average sharing of IBD blocks at the individual level. Green and red cells indicate ratios higher and lower than 1, respectively. N.S. stands for not significant Levene test for equality of variances. Abbreviations as in Table 1.

	BAS	BEN	CFT	CVV	NSA	ORK	SAP	SAU	SGL	TIM	ALB	BUL	CIT	CRO	FRA	GBR	GRE	NIT	NOR	POL	RUS	SIC	SIT	SPA
**BAS**	1.00	3.32	4.85	3.59	3.20	2.96	16.29	39.50	3.78	11.68	0.67	0.44	1.74	0.26	0.17	0.03	0.48	5.48	0.37	0.51	0.58	N.S.	N.S.	N.S.
**BEN**	0.30	1.00	1.46	N.S.	N.S.	N.S.	4.91	11.91	N.S.	3.52	0.20	0.13	N.S.	N.S.	0.05	N.S.	0.15	1.65	0.11	0.15	0.18	N.S.	N.S.	0.19
**CFT**	0.21	0.68	1.00	0.74	0.66	0.61	3.36	8.15	0.78	2.41	0.14	0.09	0.36	0.05	0.04	0.01	0.10	N.S.	0.08	0.10	0.12	0.29	0.04	0.13
**CVV**	0.28	N.S.	1.35	1.00	N.S.	N.S.	4.54	11.00	N.S.	3.25	0.19	0.12	N.S.	N.S.	N.S.	N.S.	N.S.	1.53	0.10	0.14	0.16	N.S.	N.S.	N.S.
**NSA**	0.31	N.S.	1.51	N.S.	1.00	N.S.	5.09	12.34	N.S.	3.65	0.21	0.14	N.S.	0.08	0.05	N.S.	0.15	N.S.	0.12	0.16	0.18	N.S.	N.S.	0.20
**ORK**	0.34	N.S.	1.64	N.S.	N.S.	1.00	5.51	13.36	N.S.	3.95	0.23	0.15	N.S.	0.09	0.06	0.01	0.16	N.S.	0.12	0.17	0.20	N.S.	0.07	0.21
**SAP**	0.06	0.20	0.30	0.22	0.20	0.18	1.00	2.43	0.23	0.72	0.04	0.03	0.11	0.02	0.01	0.00	0.03	0.34	0.02	0.03	0.04	0.09	0.01	0.04
**SAU**	0.03	0.08	0.12	0.09	0.08	0.07	0.41	1.00	0.10	0.30	0.02	0.01	0.04	0.01	0.00	0.00	0.01	0.14	0.01	0.01	0.01	0.04	0.01	0.02
**SGL**	0.26	N.S.	1.28	N.S.	N.S.	N.S.	4.31	10.45	1.00	3.09	0.18	0.12	N.S.	0.07	0.05	N.S.	0.13	N.S.	0.10	0.13	0.15	N.S.	N.S.	0.17
**TIM**	0.09	0.28	0.42	0.31	0.27	0.25	1.39	3.38	0.32	1.00	0.06	0.04	0.15	0.02	0.01	0.00	0.04	0.47	0.03	0.04	0.05	0.12	0.02	0.05
**ALB**	1.49	4.95	7.23	5.36	4.78	4.41	24.30	58.95	5.64	17.43	1.00	N.S.	2.60	N.S.	0.26	0.04	N.S.	8.18	0.55	0.76	N.S.	2.08	N.S.	N.S.
**BUL**	2.27	7.52	10.99	8.14	7.26	6.70	36.92	89.55	8.57	26.48	N.S.	1.00	3.95	N.S.	N.S.	N.S.	N.S.	12.43	N.S.	N.S.	N.S.	3.15	N.S.	N.S.
**CIT**	0.57	N.S.	2.78	N.S.	N.S.	N.S.	9.35	22.67	N.S.	6.70	0.38	0.25	1.00	0.15	0.10	N.S.	0.28	N.S.	0.21	0.29	0.33	N.S.	N.S.	0.36
**CRO**	3.78	N.S.	18.33	N.S.	12.11	11.18	61.61	149.41	14.30	44.17	N.S.	N.S.	6.59	1.00	N.S.	0.10	N.S.	N.S.	N.S.	N.S.	N.S.	5.26	N.S.	2.39
**FRA**	5.74	19.05	27.84	N.S.	18.39	16.98	93.54	226.87	21.71	67.07	3.85	N.S.	10.01	N.S.	1.00	N.S.	N.S.	N.S.	2.12	N.S.	3.35	7.99	N.S.	3.63
**GBR**	39.28	N.S.	190.41	N.S.	N.S.	116.11	639.81	1551.74	N.S.	458.76	26.32	N.S.	N.S.	10.39	N.S.	1.00	N.S.	N.S.	14.49	N.S.	22.88	54.64	8.37	24.80
**GRE**	2.07	6.87	10.04	N.S.	6.63	6.12	33.74	81.82	7.83	24.19	N.S.	N.S.	3.61	N.S.	N.S.	N.S.	1.00	11.35	N.S.	N.S.	N.S.	2.88	N.S.	N.S.
**NIT**	0.18	0.61	N.S.	0.66	N.S.	N.S.	2.97	7.21	N.S.	2.13	0.12	0.08	N.S.	N.S.	N.S.	N.S.	0.09	1.00	0.07	0.09	0.11	N.S.	N.S.	0.12
**NOR**	2.71	9.00	13.14	9.74	8.68	8.01	44.16	107.10	10.25	31.66	1.82	N.S.	4.72	N.S.	0.47	0.07	N.S.	14.86	1.00	N.S.	1.58	3.77	N.S.	1.71
**POL**	1.97	6.53	9.54	7.07	6.30	5.82	32.05	77.73	7.44	22.98	1.32	N.S.	3.43	N.S.	N.S.	N.S.	N.S.	10.78	N.S.	1.00	1.15	2.74	N.S.	N.S.
**RUS**	1.72	5.70	8.32	6.16	5.50	5.07	27.96	67.81	6.49	20.05	N.S.	N.S.	2.99	N.S.	0.30	0.04	N.S.	9.41	0.63	0.87	1.00	2.39	N.S.	N.S.
**SIC**	N.S.	N.S.	3.48	N.S.	N.S.	N.S.	11.71	28.40	N.S.	8.40	0.48	0.32	N.S.	0.19	0.13	0.02	0.35	N.S.	0.27	0.37	0.42	1.00	0.15	0.45
**SIT**	N.S.	N.S.	22.74	N.S.	N.S.	13.87	76.42	185.33	N.S.	54.79	N.S.	N.S.	N.S.	N.S.	N.S.	0.12	N.S.	N.S.	N.S.	N.S.	N.S.	6.53	1.00	N.S.
**SPA**	N.S.	5.25	7.68	N.S.	5.07	4.68	25.80	62.57	5.99	18.50	N.S.	N.S.	2.76	0.42	0.28	0.04	N.S.	8.68	0.58	N.S.	N.S.	2.20	N.S.	1.00

Table S7. Values of the number and total length of RoHs obtained with stringent and relaxed settings. Abbreviations as in Table 1.

	Strict settings				Relaxed settings			
	Number of RoHs		Total length of RoHs (Mb)		Number of RoHs		Total length of RoHs (Mb)	
	Average	s.d.	Average	s.d.	Average	s.d.	Average	s.d.
ALB	8.292	3.846	21.055	19.549	8.292	3.846	20.470	19.298
BAS	15.083	3.968	38.629	32.000	15.208	3.916	37.642	31.808
BEN	11.040	4.359	29.284	21.035	11.040	4.359	28.455	20.898
BUL	4.742	2.140	10.837	9.415	4.774	2.151	10.496	9.293
CFT	8.880	3.830	34.311	20.672	8.920	3.825	33.654	20.376
CIT	5.000	2.498	11.059	10.296	5.000	2.498	10.616	10.125
CRO	6.050	2.673	12.443	9.552	6.050	2.673	11.926	9.376
CVV	6.970	3.060	16.403	10.446	6.970	3.060	15.938	10.332
FRA	6.357	2.689	17.657	26.744	6.357	2.689	17.212	26.700
GBR	5.563	2.978	17.695	25.505	5.563	2.978	17.269	25.317
GRE	4.550	2.224	10.206	7.544	4.550	2.224	9.829	7.430
NIT	8.318	4.311	25.620	17.926	8.318	4.311	25.022	17.808
NOR	7.222	3.119	13.987	10.337	7.222	3.119	13.492	10.135
NSA	10.640	6.492	37.943	41.866	10.720	6.533	37.324	41.527
ORK	7.933	2.351	21.431	11.687	7.933	2.351	20.766	11.529
POL	7.188	2.888	16.035	15.300	7.219	2.880	15.487	15.195
RUS	8.040	2.630	16.567	7.478	8.040	2.630	15.828	7.335
SAP	16.125	4.521	68.042	25.945	16.167	4.515	66.935	25.814
SAU	21.200	6.400	130.786	77.773	21.300	6.198	129.503	77.237
SGL	7.783	3.243	19.295	10.975	7.783	3.243	18.804	10.895
SIC	4.850	4.053	23.187	46.046	4.950	4.177	22.918	45.962
SIT	6.000	5.364	24.778	53.381	6.000	5.364	24.250	53.095
SPA	4.794	2.153	8.764	4.650	4.824	2.135	8.326	4.461
TIM	11.042	5.152	45.899	34.455	11.167	5.297	45.206	34.222

Table S8. Effective population size estimates based on 68205 snps (16 chromosomes). Abbreviations as in Table 1.

	Population	Lower bound	Ne estimate	Upper bound
ISOLATED	BAS	3607	3739	3880
	BEN	2037	2077	2118
	CFT	2220	2266	2315
	CVV	2683	2729	2777
	NSA	2561	2623	2689
	ORK	712	720	729
	SAP	655	659	663
	SAU	208	209	210
	SGL	1717	1748	1780
	TIM	756	764	770
OPEN	ALB	2342	2396	2452
	BUL	5642	5860	6095
	CIT	5699	6009	6354
	CRO	3263	3390	3528
	FRA	6769	7190	7667
	GBR	5794	6356	7040
	GRE	3268	3396	3534
	NIT	2087	2135	2186
	NOR	3663	3848	4053
	POL	7850	8267	8732
	RUS	4821	5042	5283
	SIC	2865	2962	3065
	SIT	2660	2758	2864
	SPA	7355	7730	8143

Table S9. Estimates of current effective population size using two different methods and SNP panels. The values obtained with the IBD method for BAS and RUS refer to 333 ybp whereas for ORK and FRA to 300 ybp. Abbreviations as in Table 1.

	Current Ne estimates LD method	Current Ne estimates IBD method
BAS	3739	4712862.375
ORK	720	1604719.384
FRA	7190	5393037.629
RUS	5042	5167830.141

Supplementary Table S10. Population pairwise ratios between values of number of generations necessary to reach the observed inbreeding coefficient at any given Ne. Upper and lower bounds were obtained assuming initial inbreeding coefficients to be equal to the highest and lowest values observed among open populations, respectively.

	Lower bound	Upper bound
Basques/Sappada	0.501	0.306
Basques/Sauris	0.240	0.129
North Sardinia/Sappada	0.489	0.289
North Sardinia/Sauris	0.235	0.115

Supplementary Table S11. Multiple regression analysis performed on the entire dataset and the isolated and open population groups separately.

Principal component 1														
All populations					Isolated populations					Open populations				
Model	R	R^2	Corrected R^2	Significance	Model	R	R^2	Corrected R^2	Significance	Model	R	R^2	Corrected R^2	Significance
FRoH, Drift	0.949	0.901	0.891	0.000	FRoH	0.954	0.909	0.898	0.000	-	-	-	-	-

Principal component 2														
All populations					Isolated populations					Open populations				
Model	R	R^2	Corrected R^2	Significance	Model	R	R^2	Corrected R^2	Significance	Model	R	R^2	Corrected R^2	Significance
-	-	-	-	-	Ne	0.787	0.620	0.572	0.007	Drift	0.538	0.289	0.230	0.047
